# Loss of α-9 Nicotinic Acetylcholine Receptor Subunit Predominantly Results in Impaired Postural Stability Rather Than Gaze Stability

**DOI:** 10.3389/fncel.2021.799752

**Published:** 2022-01-13

**Authors:** Hui Ho Vanessa Chang, Barbara J. Morley, Kathleen E. Cullen

**Affiliations:** ^1^Department of Physiology, McGill University, Montreal, QC, Canada; ^2^Center for Sensory Neuroscience, Boys Town National Research Hospital, Omaha, NE, United States; ^3^Department of Biomedical Engineering, Johns Hopkins University, Baltimore, MD, United States; ^4^Department of Otolaryngology-Head and Neck Surgery, Johns Hopkins University School of Medicine, Baltimore, MD, United States; ^5^Department of Neuroscience, Johns Hopkins University School of Medicine, Baltimore, MD, United States; ^6^Kavli Neuroscience Discovery Institute, Johns Hopkins University, Baltimore, MD, United States

**Keywords:** vestibular, efferent system, vestibulo-ocular reflex, posture, balance, nicotinic

## Abstract

The functional role of the mammalian efferent vestibular system (EVS) is not fully understood. One proposal is that the mammalian EVS plays a role in the long-term calibration of central vestibular pathways, for example during development. Here to test this possibility, we studied vestibular function in mice lacking a functional α9 subunit of the nicotinic acetylcholine receptor (nAChR) gene family, which mediates efferent activation of the vestibular periphery. We focused on an α9 (−/−) model with a deletion in exons 1 and 2. First, we quantified gaze stability by testing vestibulo-ocular reflex (VOR, 0.2–3 Hz) responses of both α9 (−/−) mouse models in dark and light conditions. VOR gains and phases were comparable for both α9 (−/−) mutants and wild-type controls. Second, we confirmed the lack of an effect from the α9 (−/−) mutation on central visuo-motor pathways/eye movement pathways via analyses of the optokinetic reflex (OKR) and quick phases of the VOR. We found no differences between α9 (−/−) mutants and wild-type controls. Third and finally, we investigated postural abilities during instrumented rotarod and balance beam tasks. Head movements were quantified using a 6D microelectromechanical systems (MEMS) module fixed to the mouse’s head. Compared to wild-type controls, we found head movements were strikingly altered in α9 (−/−) mice, most notably in the pitch axis. We confirmed these later results in another α9 (−/−) model, with a deletion in the exon 4 region. Overall, we conclude that the absence of the α9 subunit of nAChRs predominately results in an impairment of posture rather than gaze.

## Introduction

The mammalian efferent vestibular system (EVS) projects from the brainstem to the inner ear and thus is theoretically well placed to modulate the afferent output of the peripheral vestibular organs (reviewed in Cullen and Wei, 2021). The somas of mammalian EVS neurons are situated within a structure called “group-e” located lateral to the abducens and genu of the facial nerve. The primary neurotransmitter of the efferent system is acetylcholine, although a diverse expression of other neuromodulators including calcitonin gene-related peptide (CGRP), 5′-triphosphate (ATP), GABA, and neuronal nitric oxide synthase (nNos) are also present at the synapse (Yamashita et al., 1993; Soto and Vega, 2010, and reviewed in Holt et al., 2010). There are two primary cholinergic receptor types found in the vestibular labyrinth: nicotinic (nAChRs) and muscarinic (mAChRs). In mammals, vestibular hair cells are characterized by α9 and α10 containing nAChRs (Hiel et al., 1996; Anderson et al., 1997; Elgoyhen et al., 2001; Luebke et al., 2005; Morley et al., 2017). When activated, α9/10 nAChRs open calcium-dependent potassium (SK) channels, which then hyperpolarizes the hair cell to inhibit neurotransmitter release (Kong et al., 2008; Turcan et al., 2010; Im, 2012).

Despite its well-understood anatomy and synaptic mechanism, the functional role of the mammalian EVS in everyday life remains poorly understood. To date, several hypotheses of functional roles of the mammalian EVS have been proposed. One longstanding view was the mammalian EVS would differentiate between active and passive movements at the periphery by transmitting motor-related signals to the vestibular periphery that modulate its head motion coding range during active movement (Goldberg et al., 2000). However, this view has not been supported by experiments in monkeys as it was found that the EVS is not involved in either extra-vestibular sensory integration or context-dependent information encoding (Cullen and Minor, 2002; Sadeghi et al., 2007; Jamali et al., 2009; Mackrous et al., 2019). It has also been proposed that the efferent vestibular system plays a slow modulatory role in shaping the functional connectivity/efficacy of the peripheral organs during maturation (Holt et al., 2010). Consistent with this idea, CGRP null mice demonstrate a substantial reduction in the efficacy of their vestibulo-ocular reflex (Luebke et al., 2014).

One of the readily available tools to investigate the functional role of the mammalian EVS is a transgenic mouse model. As noted above, α9 and α10 containing nAChRs are widely expressed in the vestibular/auditory hair cells of the vertebrate inner ear and have not reported to exist in brain (Elgoyhen et al., 1994, 2001; Hiel et al., 1996; Simmons and Morley, 1998, 2011; Morley and Simmons, 2002). Accordingly, these nAChRs have become a popular target for deletion in studies aimed at understanding the functional role of the mammalian EVS. To date, most studies of the EVS have targeted α9 containing nAChRs and quantificatied the VOR in α9 (−/−) mutants (Eron et al., 2015; Hübner et al., 2015, 2017). Moreover, the vestibular system plays a vital role in ensuring postural as well as gaze stability. However, to date, postural deficits in the α9 (−/−) mice, contrary to their VOR phenotype, have not been extensively studied (but see Tu et al., 2017). Accordingly, here we quantified both VOR and postural stability in α9 (−/−) mutant mice and compared performance to wild-type controls (Morley et al., 2017). Additionally, we compared optokinetic reflex (OKR) responses and VOR quick phases in α9 (−/−) mutant mice and their wild type counterparts. Finally, we objectively characterized postural stability during instrumented rotarod and balance beam tasks in these mice using an approach that has been previously shown to identify postural deficits in other transgenic mice (Ono et al., 2020), namely quantifying head motion using a 6D microelectromechanical systems (MEMS) module.

Overall, we did not identify deficits in the VOR of α9 (−/−) mice. Likewise, our analyses of quick phases and Optokinetic reflex (OKR) responses also suggested that the α9 (−/−) mutation did not affect central visuo-motor pathways/eye movement pathways. Importantly, however, α9 (−/−) mice did show significant impairments in performance on the balance beam and rotarod tests. Moreover, our quantitative analysis of head motion further revealed altered movement dynamics for α9 (−/−) mice as compared to their wildtype counterparts in each task, particularly in the pitch and fore-aft axis. Finally, we confirmed these later results in another α9 (−/−) model (Vetter et al., 1999) and likewise found comparable changes in the head motion in these same axis. Thus, taken together, our results suggest the loss of the α9 subunit of nAChRs largely affected postural rather than gaze stability.

## Results

### Vestibulo-Ocular Reflex and Optokinetic Reflex Responses Are Normal in α9 (−/−) Mice

**FIGURE 1:**
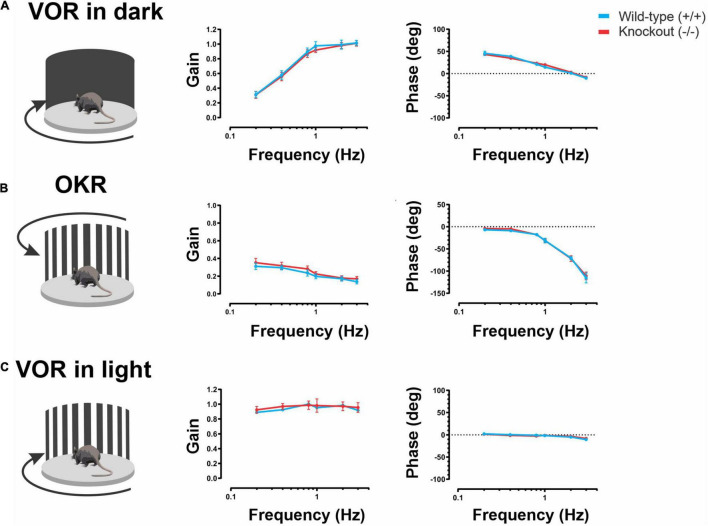
**(A)** VORd gain and phase (mean ± SEM) plotted as a function of frequency for wild-type and α9 (–/–) mice. N is 7 and 8 for both wild-type (blue) and α9 (–/–) (red) mice, respectively, for the model with a deletion in exon 1 and 2. **(B)** OKR gain and phase (mean ± SEM) plotted as a function of frequency wild-type and α9 (–/–) mice. **(C)** VORl gain and phase (mean ± SEM) plotted as a function of frequency for wild-type and α9 (–/–) mice.

We first measured VOR responses in α9 (−/−) mice and their wild-type counterparts (Morley et al., 2017). We applied sinusoidal rotations at 0.2, 0.4, 0.8, 1, 2, and 3 Hz with a peak velocity of 16°/s. VOR responses were tested in the dark (referred to as VORd). The gain and phase of the eye movements generated during VORd testing were then quantified for each frequency of rotation. The robust compensatory eye movements generated by wild-type mice during VORd testing increased with frequency reaching fully compensatory gains (∼unity) for stimulation ∼0.8 Hz and higher (Figure 1A, blue trace). Similarly, α9 (−/−) mice generated robust compensatory eye movements across this frequency range. Indeed, VORd gains and phases were comparable to those of wild-type mice (Figure 1A, compare red and blue traces; *P* > 0.5, *P* > 0.5).

The optokinetic reflex (OKR) is a visually driven reflex that generates eye movements in response to a moving visual scene. During self-motion, the OKR complements the VOR to minimize visual motion on the retina to provide gaze stability at lower frequencies (reviewed in Cullen, 2019). Notably, the OKR shares premotor circuitry with the VORd (for review, see Cullen and Van Horn, 2011). Thus, we measured OKR responses as a control for testing whether there are any deficits resulting from non-vestibular effects in the VOR circuitry. During OKR testing, the visual surround was rotated sinusoidally using the same stimulus as that used for VORd testing (i.e., 0.2, 0.4, 0.8, 1, 2, and 3 Hz; peak velocity of 16°/s). Quantification of the eye movements evoked by OKR testing revealed no difference in the response gains or phases of α9 (−/−) mice compared to their wild-type counterparts (Figure 1B; *P* > 0.5, *P* > 0.5).

Finally, during actual self-motion in the light, both the VOR and OKR work synergistically to stabilize gaze (reviewed in Cullen, 2019). Accordingly, we applied sinusoidal rotations at 0.2, 0.4, 0.8, 1, 2, and 3 Hz with a peak velocity of 16°/s. VOR responses in the light (referred to as VORl). Consistent with the lack of difference found during VORd and OKR testing, quantification of the eye movements evoked by VORl testing revealed no difference in the response gains or phases of α9 (−/−) mice compared to their wild-type counterparts (Figure 1C; *P* > 0.5, *P* > 0.5). Thus, taken together, our results demonstrate that the loss of the α9 subunit of nAChRs did not alter gaze stability in the frequency range between 0.2 and 3 Hz.

### Quick Phase Eye Movement Dynamics Are Not Changed in α9 (−/−) Mice

**FIGURE 2:**
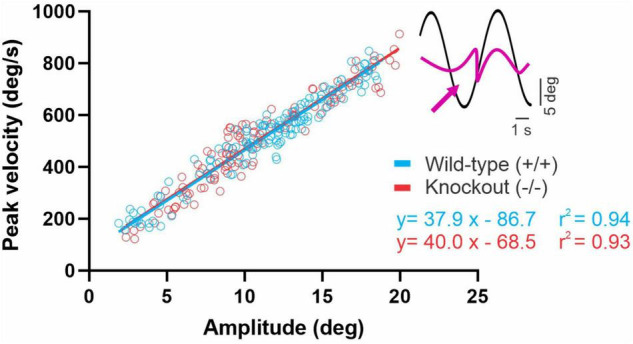
Peak velocity vs. amplitude relationship for quick phases of the VOR of wild-type and α9 (–/–) mice. Regression lines are superimposed. There is no significant difference between the two slopes.

To further investigate whether eye movement control was altered in α9 (−/−) mice, we next quantified the relationship between the peak velocity and amplitude of quick phases evoked during rotational stimulation (i.e., main sequence relationship). In agreement with previous studies, this relationship was linear for our wild-type mice (Figure 2, blue symbols; *R*^2^ = 0.94). Similarly, a similarly robust linear relationship was also observed for α9 (−/−) mice (Figure 2, red symbols; *R*^2^ = 0.93). Furthermore, the slope of the main sequence relationship in α9 (−/−) mice and their wild-type mice counterparts were comparable (slope 37.9 versus 40.0, respectively; *P* = 0.0978). Thus, together with the results in Figure 1 above, our findings suggest that the loss of α9 loss does not alter vestibularly driven VOR eye movements, visually driven OKR eye movements, stabilizing eye movements generated by the integration of the vestibular and visual inputs, or quick phase eye movements.

### α9 (−/−) Mice Show Impaired Postural Control on a Rotarod

**FIGURE 3:**
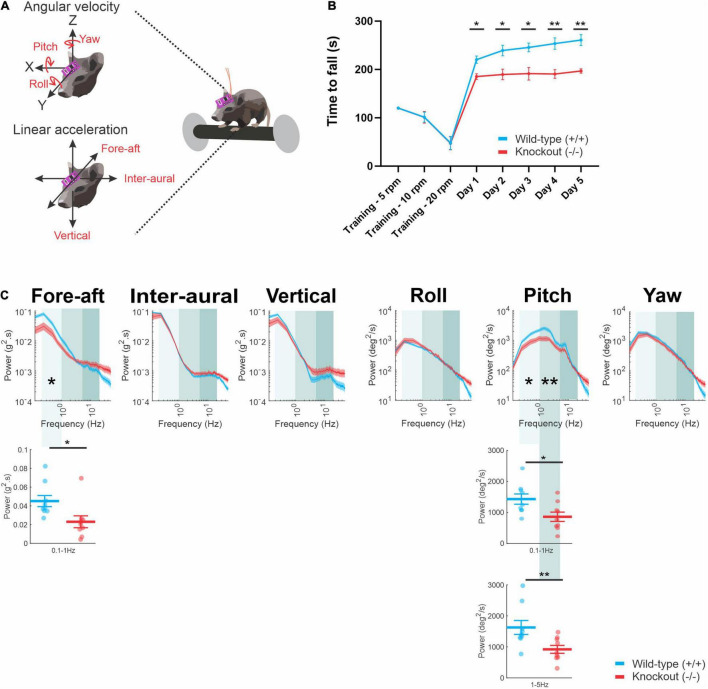
**(A)** A schematic of an accelerating rotarod assay. **(B)** Time to fall measured each day (mean ± SEM) for wild-type (blue) mice and α9 (–/–) mice (red) **(C)** Comparison of power spectra density of head movements during rotarod in translational axis and rotational axis between wild-type mice and α9 (–/–) mice. N is 9 and 9 for both wild-type and α9 (–/–) mice. Error bars: SEM. *P < 0.05, **P < 0.01.

As noted above, to date, whether the loss of α9 results in postural deficits has not been well studied. Accordingly, we next tested whether α9 (−/−) mice demonstrate any postural impairments. We first conducted standard behavioral testing that included: (1) tail hanging test, (2) air righting, (3) contact inhibition of righting, and (4) swimming. All mice, regardless of their genotype, scored 0 (normal) on all listed behavioral tests (data not shown). We then subjected mice to an accelerating rotarod (Figure 3A). All mice were subjected to three training sessions with three different constant rpm settings (at 5, 10, and 20 rpm). During the testing sessions, the rotarod was set to increase its rpm from 4 to 40 in 300 s. The time to fall from the rotarod was used to quantify their performance. The trial was repeated for five consecutive days. Overall, we found that α9 (−/−) mice showed poorer performance than their wild-type counterparts. Specifically, α9 (−/−) mice fell significantly earlier from the first day of testing (day 1) to the last day (day 5) (Figure 3B) (day 1, *P* = 0.0183; day 2, *P* = 0.0435; day 3, *P* = 0.0390; day 4, *P* = 0.00481; day 5, *P* = 0.0011).

### α9 (−/−) Mice Show Different Head Movement Dynamics Compared to Wild-Type Mice on a Rotarod

Given the poorer performance of α9 (−/−) mice on a rotarod, we hypothesized that α9 (−/−) mice should exhibit different head movement dynamics while balancing on the rotarod compared to wild-type mice. Accordingly, we quantified their head motion using a 6D MEMS module consisting of three gyroscopes and three linear accelerometers affixed to the animal’s head post. All head movement was collected during day 5 of testing. Power spectra of head movements revealed significantly lower power in α9 (−/−) mice as compared to their wild-type counterparts in the fore-aft and pitch axis at lower (0.1–1 Hz) frequencies (Figure 3C, compare red and blue traces; fore-aft, *P* = 0.021; pitch, *P* = 0.021). Significantly lower power was also found in the pitch axis in the mid (e.g., 1–5 Hz) frequencies range (pitch, *P* = 0.0064). Thus, together, the altered head movement dynamics of α9 (−/−) mice in the fore-aft and pitch axis coupled with their and poorer scored performance indicates that their ability to maintain balance while completing the task was impaired. We further consider this point below in the section “Discussion.”

### α9 (−/−) Mice Are Slower and Show Different Head Movement Dynamics Compared to Wild-Type Mice on a Balance Beam

**FIGURE 4:**
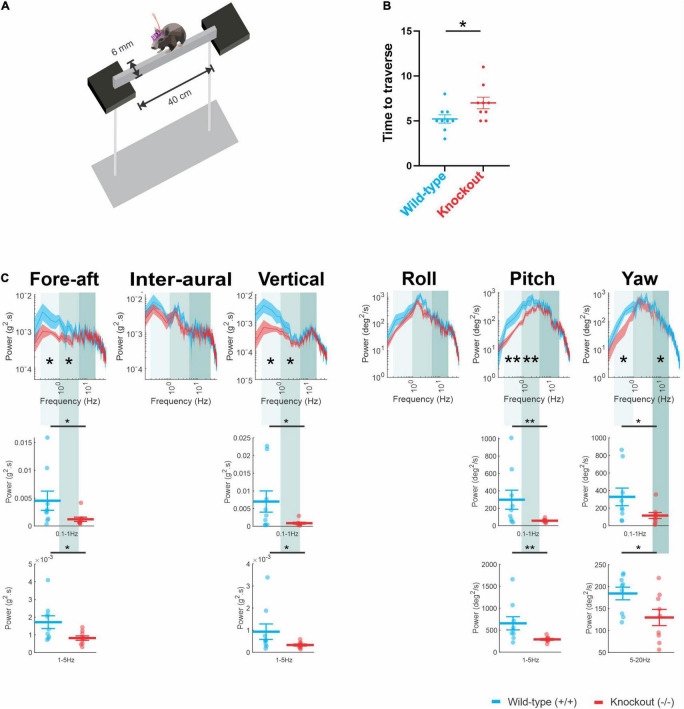
**(A)** A schematic of a narrow balance bean assay. **(B)** Time taken to traverse the balance beam (mean ± SEM) for wild-type mice (blue) and α9 (–/–) mice (red) **(C)** Comparison of power spectra density of head movements during balance beam in translational axis and rotational axis between wild-type mice and α9 (–/–) mice. N is 9 and 9 for both wild-type and α9 (–/–) mice. Error bars: SEM. *P < 0.05, **P < 0.01.

To further test whether the loss of α9 results in postural deficits, we tested the ability of our mice on a challenging balance test in which they locomoted across a narrow 6-mm balance beam (Figure 4A). Overall, we found that the performance of our α9 (−/−) mice were markedly impaired during this task. Notably- α9 (−/−) mice took significantly longer to cross the balance beam compared to their wild-type counterparts (Figure 4B; *P* = 0.0322). Accordingly, this result, together with our above results quantifying performance on the rotarod, is further indicative of impaired balance in α9 (−/−) mice.

As α9 (−/−) mice generally showed lower power in fore-aft and pitch axis while balancing on the rotarod, we hypothesized that mouse head movement dynamics on the balance beam would exhibit similar trends. Accordingly, we measured head movement in mice while they traversed the narrow 6-mm balance beam. Indeed, our power spectra analysis of the head motion generated during this testing revealed lower power in the low-frequency range (fore-aft, *P* = 0.041; vert, *P* = 0.021; pitch, *P* = 0.0067; yaw, *P* = 0.05) and mid and high-frequency ranges (mid-frequency: 1–5 Hz; vert, *P* = 0.039; pitch, *P* = 0.0028; High frequency: yaw, *P* = 0.035) (Figure 4C). This consistent pattern of lower power in fore-aft and pitch axis coupled with longer crossing times is consistent with the observed impairment in the ability of α9 (−/−) mice to maintain postural stability during rotarod testing. We further discuss the implications of these results below in the section “Discussion.”

### Exploratory and Resting Head Movement Dynamics Are Comparable for α9 (−/−) Mice and Wild-Type Mice

Given that the mutant mice took longer to cross the balance beam (Figure 4 above) we next investigated whether this might be explained by hypo-activity. Notably, our subjective visual inspection of these mice in the cage did not reveal any signs of hypo-activity. In addition, we placed both groups of mice within a cylinder and objectively quantified their activity levels, using two approaches. First, we quantified the number of times that the mice touch the wall of cylinder. We found no difference between two groups. Secondly, we plotted and compare power spectra of the animal’s head movement during their time within the cylinder and found no significant differences in any motion dimension. Thus, these results suggest that impaired balance rather than hypo-activity in knockout mice led to longer transverse time on the balance beam.

Additionally, it is noteworthy that mutant mice strain with vestibular dysfunction can demonstrate head movement tremors/oscillations while at rest (see for example, Ono et al., 2020). To rule out the possibility the significant differences in head movement dynamics observed during balance beam and rotarod testing were due to the difference that is already present at rest, we reanalyzed the head movement data recorded when the mouse was placed in a cylinder, focused solely on epochs of time where mice (i) did not make exploratory head movement, and (ii) where their bodies remained stationary. No difference was observed in any axis between α9 (−/−) and control wild-type mice, indicating the lack of significant head tremors/oscillations while at rest in α9 (−/−) mice. Taken together, these results further strengthen our conclusion that postural stability is impaired in α9 (−/−) mice.

### Postural Phenotypes Are Similarly Altered in a Second α9 (−/−) Mouse Model

**FIGURE 5:**
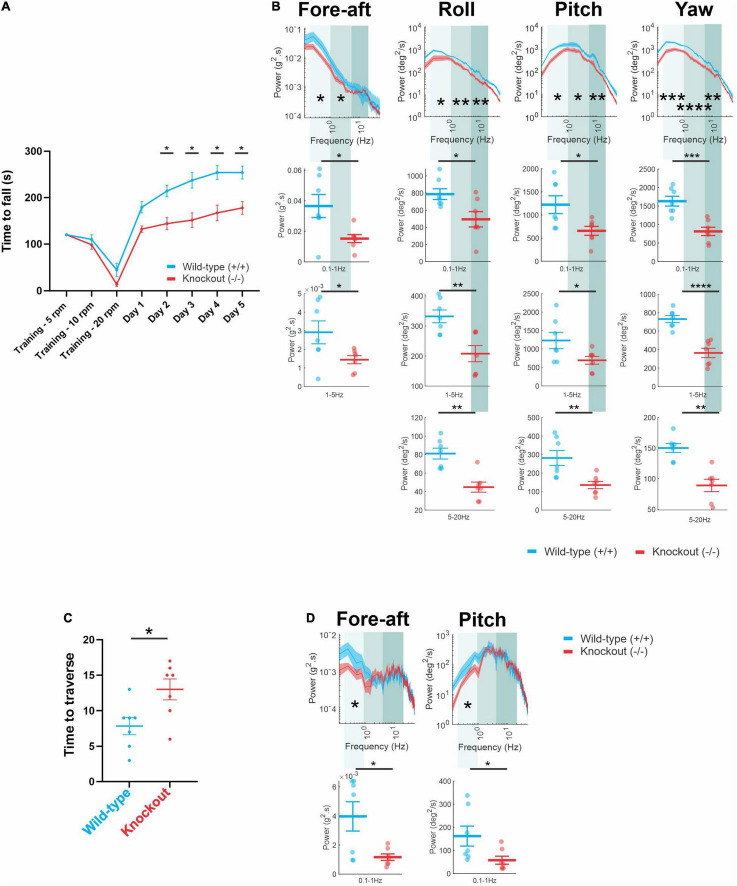
**(A)** Time to fall measured each day (mean ± SEM) for wild-type mice (blue) and α9 (–/–) mice (red). **(B)** Comparison of power spectra density of head movements during rotarod in fore-aft, roll, pitch, and yaw axis between wild-type mice and α9 (–/–) mice. **(C)** Time taken to traverse the balance beam (mean ± SEM) for wild-type mice and α9 (–/–) mice. **(D)** Comparison of power spectra density of head movements during balance beam in fore-aft and pitch axis between wild-type mice and α9 (–/–) mice. N is 7 and 7 for both wild-type and α9 (–/–) mice. Error bars: SEM. *P < 0.05, **P < 0.01, ***P < 0.001, ****P < 0.0001.

Finally, because the question of whether the loss of α9 results in postural deficits had not been well investigated prior to this study, we also completed the same rotarod and balance beam testing shown above in Figures 3, 4 in another α9 (−/−) mouse model (Vetter et al., 1999, see section “Materials and Methods”). Similar to our results above, we found that these α9 (−/−) mice also fell significantly earlier than their wild-type counterparts, beginning on day 2 to day 5 (Figure 5A; day 2, *P* = 0.0157; day 3, *P* = 0.0218; day 4, *P* = 0.0186; day 5, *P* = 0.0155). Furthermore, a comparable quantification of 6-dimensional head motion during the rotarod test (Figure 5B) revealed that these α9 (−/−) mice likewise demonstrated a reduction in power in the fore-aft and pitch axis in both the low (fore-aft, *P* = 0.017; pitch, *P* = 0.025) and mid (fore-aft, *P* = 0.05; pitch, *P* = 0.045) frequency ranges, and significant differences were further found in this range for the roll and yaw axis (low frequency: roll, *P* = 0.018; yaw, *P* = 0.00075; mid-frequency: roll, *P* = 0.0058; yaw, *P* = 0.00045). Moreover, differences were seen in several axis in the high-frequency range (roll, *P* = 0.0019; pitch, *P* = 0.0038; yaw, *P* = 0.0011). Correspondingly, we found that these same α9 (−/−) mice also took significantly longer to transverse the narrow balance beam compared to their wild-type counterparts (Figure 5C; *P* = 0.023) and showed lower head motion power in fore-aft and pitch axis for the low-frequency range (Figure 5D; fore-aft, *P* = 0.037; pitch, *P* = 0.041). Thus, overall, the loss of α9 resulted in similar postural deficits in both α9 (−/−) mouse models tested in our study.

## Discussion

In this study, we tested the vestibular function of mice lacking the α9 subunit of nicotinic acetylcholine receptors (nAChRs). Specifically, we tested both VOR and postural stability in an α9 (−/−) mouse model developed by Morley et al. (2017). We first quantified vestibular function by measuring the eye movements generated by the vestibulo-ocular reflex (VOR), which functions to ensure gaze stability during head motion (reviewed in Goldberg et al., 2012). The gain and phase of the compensatory VOR eye movements were quantified during rotations in both dark and light conditions. We found that VOR function was unimpaired in α9 knockout mice as compared to wild-type controls. Likewise, analysis of the relationship between quick phase amplitude and velocity evoked during rotational stimulation revealed a robust linear relationship that was unchanged from their wild-type counterparts. We also quantified the gaze stabilizing eye movements generated in response to a moving visual (versus vestibular) surround [optokinetic reflex (OKR) responses]. We found that OKR response gains and phases were comparable to those measured in wild-type controls, indicating that VOR pathway function was not altered at subsequent levels of central processing.

In contrast, our analysis of postural stability and motor control revealed deficits in these same α9 (−/−) mice. Notably, we scored performance during three tasks: standard rotarod testing, the balance beam test, and swimming test. In addition, head movements were quantified using a 6D microelectromechanical systems (MEMS) module fixed to the animal’s head during rotarod testing, balance beam testing, and for resting head movements when the animal was stationary. Overall, we found impaired performance during rotarod testing; the time to fall was shorter for α9 (−/−) mice as compared to their wild-type counterparts. Likewise, we found impaired performance during balance beam testing; the crossing time was longer for α9 (−/−) mice. Furthermore, quantitative analysis of head motion during rotarod and balance beam testing consistently revealed altered head motion power – including a decrease in fore-aft and pitch axis rotation. Thus, overall, the ability of α9 (−/−) mice was impaired during both tasks, and in turn, head movement power was lower in α9 (−/−) mice compared to their wild-type counterparts. We further confirmed these later results in a second α9 (−/−) model (Vetter et al., 1999). Our results suggest that mutant mice were more restrained in their movements due to compromised vestibular function, and as such their ability to generate robust compensatory movements to remained balanced was reduced relative to wild-type mice. We speculate that the most significantly impacted dimensions of motion (i.e., fore-aft and pitch axis rotation) corresponded to those for which the neural/biomechanical challenge to the balance system was the greatest during rotarod and balance beam tasks. Thus, taken together, our results provide strong evidence that the absence of the α9 nAChR subunit predominately results in impairments in postural rather than gaze stability.

### Relationship to the Existing Vestibulo-Ocular Reflex Literature

Prior to the present study, our understanding of how the loss of the α9 subunit of the nicotinic acetylcholine receptor (nAChRs) alters vestibular function had been based largely on the measurement and quantification of VOR responses. Here, we first reported the VOR and postural responses of the same α9 knockout mice (Morley et al., 2017). We found that these mice show no difference in their vestibular driven eye movements as compared to wild-type controls over the frequency range tested (rotations up to 3 Hz). The gains and phases of the compensatory VOR were unchanged in α9 (−/−) mice. Notably, this result is not consistent with those of previous studies reporting significantly reduced gain of the compensatory VOR evoked by stimulations >1 Hz (Hübner et al., 2015). One possible difference is the genetic background of the mice used in our studies and in the Hübner studies. It is not uncommon for a gene on different genetic backgrounds to produce a different phenotype. The mice used in the studies reported here are on C57BL/6J mice while the Hübner studies were obtained from The Jackson Laboratory (JAX) and originated from the mice described by Vetter et al. (1999). The mice were originally on a CBA/CaJ X 129Sv background and maintained on an equivalent mixed strain. Additionally, Hübner et al. (2015) used an independent colony of hybrid CBA/CaJ x 129/SvEv as their control rather than using litter-mate controls, which could also potentially contribute to the differences reported in this study.

In addition, our analysis of the VOR quick phases responses (i.e., main sequence analysis) revealed that no change in the eye movements generated by α9 knockout mice as compared to their wild-type controls, consistent with (Hübner et al., 2015, but see Eron et al., 2015). In addition to ACh, the neuroactive peptide calcitonin-gene-related peptide (CGRP) acts at efferent synapses. Thus, it is noteworthy that, in contrast to α9 knockout mice, CGRP knockout mice demonstrate a profound (50%) reduction in the efficacy of their vestibulo-ocular reflex (Luebke et al., 2014). It has thus been proposed that the CGRP may play a slow modulatory role in shaping the functional connectivity/efficacy of the VOR pathways during maturation.

### α9 and the Efferent Vestibular System: Implications for Vestibular Pathway Modulation

The cell bodies of the mammalian efferent vestibular system are located in the group-e nucleus, situated just dorsal to the genu of the facial nerve and just medial to the VI (abducens) nucleus. Stimulation of the mammalian “group-e” nucleus evokes excitatory responses in both ipsilateral and contralateral vestibular afferents (Goldberg and Fernandez, 1980; McCue and Guinan, 1994; Marlinski et al., 2004), characterized by fast and slow responses with activation time constants of 100 ms versus seconds, respectively (Goldberg and Fernandez, 1980; Sadeghi et al., 2009). Acetylcholine (ACh) is the primary neurotransmitter at the efferent vestibular synapses across vertebrate classes (reviewed in Holt et al., 2010; Goldberg et al., 2012). In mammals, nicotinic ACh receptors (nAChRs) are found in the vestibular labyrinth (Hiel et al., 1996; Elgoyhen et al., 2001; Luebke et al., 2014), and the efferent-mediated release of ACh activates these α9/α10 nicotinic AChRs (reviewed in Jordan et al., 2013; Poppi et al., 2020).

Mammalian vestibular afferent fibers originating in both the semicircular canals and otoliths vary in their resting discharge regularity and are commonly characterized as either “regular” or “irregular” (reviewed in Cullen, 2019). Recent studies have established that regular and irregular afferents provide two parallel streams of sensory input to mammalian central vestibular pathways (reviewed in Cullen, 2019). Regular afferents better encode information about head motion compared to irregular afferents (Sadeghi et al., 2007), whereas irregular afferents are more dynamic and better discriminate between head motion stimuli using precise (∼6 ms) spike timing (Jamali et al., 2016, 2019). Importantly, stimulation of neurons in the mammalian “group-e” nucleus evoked markedly greater responses in irregular than regular afferents (reviewed in Cullen and Wei, 2021). Furthermore, the application of nicotinic and muscarinic AChR antagonists preferentially block the fast versus slow components of the excitatory efferent-mediated responses evoked in these afferents (Schneider et al., 2021).

In this context, it is noteworthy that vestibular afferents with more irregular resting discharges (i.e., irregular afferents) are more likely to project to central vestibular nuclei neurons that comprise the vestibulo-spinal pathways, whereas those with more regular resting discharges (i.e., regular afferents) are more likely to project to central vestibular nuclei neurons that comprise the pathway generating the VOR (Goldberg et al., 1987; Sato and Sasaki, 1993). This difference in projections is consistent with studies showing that the VOR is unchanged following functional ablation of irregular afferents (Minor and Goldberg, 1991; Angelaki and Perachio, 1993). Thus, given that experimental activation of the efferent vestibular system (EVS) produces a markedly larger effect on the responses of irregular than regular afferents, one logical hypothesis is that the EVS play a more significant role modulating vestibulo-spinal vs. vestibulo-ocular reflex pathways. Interestingly, this proposal is consistent with our present results, where we found significant differences in the postural performance of two distinct α9 (−/−) mouse models, while VOR eye movements for stimulation up to 3 Hz were not altered.

As noted above prior studies have reported a significant reduction of VOR gain in α9 (−/−) mice for higher frequencies of stimulation (up to 10 Hz; Hübner et al., 2015). Additionally, this same group reported that the adaptation of the VOR that normally occurs in mice following visual-vestibular motor learning is significantly impaired in α9 (−/−) mice. Indeed, while there are reasons to believe that while regular afferents make the primary contribution to the VOR at lower frequencies of stimulation, the irregular afferents are important for VOR motor learning. In particular, the results of human and monkey studies have led to the proposal that a central pathway comprising phasic neurons, which predominately receive irregular afferent input, is highly modifiable and thus mediates visually induced VOR motor learning (Clendaniel et al., 2001, 2002) as well as compensation following peripheral vestibular loss (Lasker et al., 1999, 2000). In contrast, a central pathway comprising more tonic neurons that predominately receive regular afferent input mediates the direct unmodified VOR. Additionally, the influence of irregular afferents on the direct unmodified VOR itself should become more significant at higher frequencies. This is because the high-pass nature of their response dynamics will result in increasing larger gains compared to their regular counterparts. Given that most central neurons receive a mix of regular and irregular inputs (Goldberg et al., 1987; Sato and Sasaki, 1993), the relative contribution of irregular would then increase for VOR and vestibulo-spinal pathways as a function of frequency. Thus, it is possible that further testing with higher frequency stimulation (>3 Hz) may reveal a significant reduction of VOR in the α9 (−/−) mice that were not the focus of the present study.

### Specificity of α9 to the Periphery and Directions for Future Research

There is much evidence that the cerebellum plays an essential role in ensuring balance (reviewed in Cullen, 2019) and that intact VOR learning in mice is dependent on cerebellar innervation of brain stem vestibular nuclei (Beraneck et al., 2004). In this context, it is noteworthy that a recent study Lykhmus et al. (2017) reported that α9 transcript may be present in mitochondria in cells in the brain, most notably in the vestibular nuclei and cerebellum. The possibility of a contribution of these cells cannot currently be excluded, although methodological issues regarding these studies have been raised (Morley et al., 2018). Indeed, our present findings that OKR responses are normal in α9 (−/−) mutant models demonstrated a lack of non-vestibular deficits at the level of the oculomotor circuitry. Notably, OKR responses are produced by a premotor circuit which involved the cerebellum (for review, see Cullen and Van Horn, 2011). Thus, our finding that OKR response gains and phases were comparable for both α9 (−/−) mouse models and their control mice is consistent with the general consensus that α9 is not present in the brain (Elgoyhen et al., 1994; Morley et al., 2018). Finally, it is noteworthy that the present focused on the role of the EVS on reflex and motor function. Recent studies have demonstrated impairments in multisensory attention in α9 (−/−) mice during a visual selective attention task with auditory distractors (Terreros et al., 2016; Jorratt et al., 2017). It is possible that the EVS likewise contributes to higher-order functions, for example reducing impulsivity during decision making during self-motion. Further studies are required to test this interesting possibility.

## Materials and Methods

### Mice and Genotyping

We focused on two distinct α9 (−/−) models, with a deletion of exons 1 and 2 (*Chrna9*^TM 1Bjmy^, MGI:5787807, Morley et al., 2018) and the region of exon 4 (*Chrna9*^TM 1Bev^, MGI:2158742, Vetter et al., 1999) of the *Chrna9* gene. The α9 nAChR KO mouse model with exons 1 and 2 deleted was generated by (Genoway, Inc., Lyon, France) and was back-crossed at the Boys Town National Research Hospital (BTNRH) to C57Bl/6J mice. For the studies reported here, mice were bred and housed at BTNRH until weaned and then transported to Johns Hopkins University. The BTNRH IACUC approved the transport and use of the animals for the studies reported here. Mice with the deletion of the exon 4 region, originally reported by Vetter et al. (1999), were obtained from The Jackson Laboratory and backcrossed to the C57Bl/6J mice at Johns Hopkins University. All animal experiments were conducted under the approved animal protocols at McGill University and Johns Hopkins University. The experimenter was blind to genotype during data collection as well as analysis for each of the tests detailed below.

### Quantification of Vestibulo-Ocular Reflex and OKR

Surgical techniques and experimental setup have been previously described (Beraneck and Cullen, 2007). Eye movement data were collected using an infrared video system (ETL-200, ISCAN system). The rotational velocity of the turntable (head velocity) was measured using a MEMS sensor (MPU-9250, SparkFun Electronics). Eye movements during OKR were evoked by sinusoidal rotations of a visual surround (vertical black and white stripes, visual angle width of 5°) placed around the turntable at frequencies 0.2, 0.4, 0.8, 1, 2, and 3 Hz with peak velocities of ±16°/s. To record VOR responses, the turntable was rotated at sinusoidal frequencies 0.2, 0.4, 0.8, 1, 2, and 3 Hz with peak velocities of ±16°/s in both light and dark. In the light condition, the visual surround remained stationary, whereas, in the dark condition, both the visual surround and turntable rotated in phase. Head and eye movement signals were low-passed filtered at 125 Hz, and sampled at 1 kHz. Eye position data were differentiated to obtain velocity traces. Cycles of data with quick phases were excluded from the analysis. Least-square optimization determined the VOR and OKR gains and phases plotted as mean ± standard error of the mean (SEM) against all frequencies for all mice.

### Rotarod

Mice were trained with trials of 5, 10, and 20 rpm for 120 s each with 15 min rest periods between trials and a 1-min acclimation period. Mice were allowed to acclimate for 5 min on the rotarod during training and test trials. For each test trials, the rotarod accelerated from 4 rpm to 40 rpm with a ramp of 300 s.

Head movements in six dimensions were also recorded using a miniature head motion sensor (MPU-9250, SparkFun Electronics, Niwot, CO, United States) affixed on the top of the skull, which comprises a three-dimensional (3D) accelerometer (measures linear acceleration; fore/aft, lateral, and vertical) and 3D gyroscope (measures angular velocity: roll, pitch, and yaw). Data was acquired at 200 Hz using windows-based CoolTerm software. We then computed the power spectral densities (pwelch function, MATLAB, MathWorks) using Welch’s averaged periodogram with nfft = 4,096 and a Bartlett window (4,096 ms duration) for all six dimensions of movement.

### Balance Beam

A 6-mm-wide and 40-cm-long beam was used for balance beam testing. Mice traverse 40 cm. Walking speed was measured by recording the time the mouse took to reach the goal box from the opposite end of the beam. Mice were scored “time out” when failed to reach the endpoint in 2 min. Head movements in six dimensions were also recorded (See section “Rotarod”).

### Air Righting, Tailing Hanging, and Contact Inhibition of Righting Tests

#### Air Righting Test

Mouse was picked up by the tail and lowered into a container so that all four feet were touching the bottom. The container was quickly inverted at a height of 30–40 cm so mouse fell supine. How mouse lands onto a foam cushion were observed and rated as below (Rabbath et al., 2001):

• 0 = the animal lands on its feet (normal).
• 1 = the animal lands on its side (mild deficit).
• 2 = the animal lands on its back (severe deficit).

#### Tail Hanging Test

Mouse was picked up by the tail and lowered to an even surface. Mouse’s posture was rated using the following scale (Rabbath et al., 2001):

• 0 = straight body posture with extension of forelimbs toward the earth (normal).
• 1 = slightly bending the body ventrally (intermediate response).
• 2 = persistently bending the body (severe response).

#### Contact Inhibition of Righting Test

Mouse was picked up by the tail and lowered into a container so that all four feet are touching the bottom. The container was quickly inverted so the mouse was supine while a surface was touching the soles of the mouse’s feet. The mouse’s reflex was rated using the following scale (Rabbath et al., 2001):

• 0 = animal rights successfully (normal).
• 1 = partial righting (intermediate response).
• 2 = complete loss of righting (severe response).

### Swimming

A large container (26.25 × 16.25 × 14.38′) was filled with water (24–26°C) at the height of at least 15 cm. The mouse was placed into the water and observed for up to 1 min. Its preformation was rated using the following scale (Hardisty-Hughes et al., 2010):

• 0 = swims, body elongated, and tail propels in flagella-like motion.
• 1 = immobile floating.
• 2 = underwater tumbling.

### Resting Head Movement

Mice were placed in a cylinder (9 cm diameter and 21.5 cm height) that limited their motion, so mice maintained their steady posture. Head movements in six dimensions were recorded (See section “Rotarod”).

### Statistical Analyses

Data are reported as the mean ± SEM. Non-parametric Mann–Whitney *U*-test was performed to test significance for time to traverse (balance beam) between two groups. Two-way repeated-measures ANOVA followed by Bonferroni *post hoc* comparison tests was used for the following data: (1) the VOR data across frequencies and; (2) the latency to fall off the rotarod for 5 consecutive days. Non-normal distributed data were transformed to [Log (X 1)] to meet the requirements of the ANOVA model. For the main sequence analysis, the extra sum-of-squares *F* test was used to compare two slopes. For power spectra analysis, we used an independent sample permutation test to test significant differences between the two groups. Prism 9 (GraphPad) or MATLAB was used for statistical analyses.

## Data Availability Statement

The raw data supporting the conclusions of this article will be made available by the authors, without undue reservation.

## Ethics Statement

The animal study was reviewed and approved by the Johns Hopkins University Animal Care and Use Committee. Written informed consent was obtained from the owners for the participation of their animals in this study.

## Author Contributions

KC and HC designed the study and wrote the manuscript in consultation with BM. HC performed the experiments, derived the models, and analyzed the data. BM developed the mouse model. All authors contributed to the article and approved the submitted version.

## Conflict of Interest

The authors declare that the research was conducted in the absence of any commercial or financial relationships that could be construed as a potential conflict of interest.

## Publisher’s Note

All claims expressed in this article are solely those of the authors and do not necessarily represent those of their affiliated organizations, or those of the publisher, the editors and the reviewers. Any product that may be evaluated in this article, or claim that may be made by its manufacturer, is not guaranteed or endorsed by the publisher.
